# Single photon triggered dianion formation in TCNQ and F_4_TCNQ crystals

**DOI:** 10.1038/srep28510

**Published:** 2016-06-27

**Authors:** Lin Ma, Peng Hu, Hui Jiang, Christian Kloc, Handong Sun, Cesare Soci, Alexander A. Voityuk, Maria E. Michel-Beyerle, Gagik G. Gurzadyan

**Affiliations:** 1 Division of Physics and Applied Physics, School of Physical and Mathematical Sciences, Nanyang Technological University, 637371 Singapore; 2 School of Materials Science and Engineering, Nanyang Technological University, Singapore 639798, Singapore; 3 Instituciό Catalana de Recerca i Estudis Avançats (ICREA), Barcelona 08010, Spain; 4 Institut de Química Computacional i Catàlisi (IQCC), Universitat de Girona, Campus de Montilivi 17071 Girona, Spain; 5 Institute of Artificial Photosynthesis, State Key Laboratory of Fine Chemicals F-209, Dalian University of Technology, Dalian, 116024, China

## Abstract

Excited state dynamics in two strong organic electron acceptor systems, TCNQ and F_4_TCNQ single crystals, was studied. After absorption of a single photon, dianions are formed in both crystals on ultrashort timescale: TCNQ τ < 50 fs, F_4_TCNQ τ = 4 ps. By use of transient absorption spectroscopy, we demonstrate that the dianion formation in F_4_TCNQ is mediated by the radical anion precursor which is described by a two-step model. Our measurements show the phenomenon that in this quinoid acceptor crystals in the absence of additional donor molecule, it is possible to resolve the two step formation of a doubly charged anion upon absorption of a single low energy photon (2.6 eV).

7,7,8,8-Tetracyanoquinodimethane (TCNQ) is a strong organic electron acceptor, with a high electron affinity of 2.8 eV^1^,^2^–3.38 eV^3^. Therefore, TCNQ has been used for synthesis of large number of charge transfer compounds that have been widely explored as building blocks of molecular electronics^4^,^5^, nonlinear optics^6^, and organic semiconductors^7^,^8^. A tetrafluorinated derivative of TCNQ, F_4_TCNQ (2,3,5,6-Tetrafluoro-7,7,8,8-tetracyanoquinodimethane), achieves an even higher electron affinity of 5.2 eV due to the presence of the four fluorine atoms^9^. The fluoride substitutes change the electrostatic potential but keep the same molecular geometry as that of TCNQ^10^. The charge transfer compounds of TCNQ and F_4_TCNQ show unusual electric and magnetic properties, ranging from metallic conductors, semiconductors to insulators^11^,^12^,^13^. After electron uptake from donor molecules, TCNQ and F_4_TCNQ are reduced to negative charged anions. These anions also classified as radical anions, TCNQ^•−^ and F_4_TCNQ^•−^, are among the very few anions which possess bound excited electronic states^14^,^15^. In crystals these bounded excitons can decay back to neutral molecules, dissociate to conducting electrons and holes or undergo a subsequent chemical reaction forming dianions from anions by further electron reduction after applying voltage^16^ or in the gas phase by collision with the sodium vapor^17^. It is noteworthy that in order to form the anion or dianion of TCNQ or F_4_TCNQ, other molecules are necessarily used as donors. These additional donors may be other molecules as tetrathiafulvalene (TTF) in charge transfer compound TTF-TCNQ or, as shown in this paper, neutral TCNQ or F_4_TCNQ. The last reaction is highly unusual. In the TCNQ and F_4_TCNQ crystals only few molecules are excited from the ground state and neighboring molecules may serve as electron donors leading to the formation of a radical ion pair according to reaction 1 when exciting the TCNQ crystal:


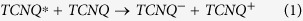


The driving force for this reaction is largely determined by the photon energy absorbed by TCNQ molecules in the crystal lattice. However, the anion of TCNQ^−^ is chemically unstable. It may recombine back to two neutral TCNQ molecules in their electronic ground states through reaction with a cation TCNQ^+^ emitting phonon or it can be further reduced by electron uptake from an adjacent neutral TCNQ molecule according to the following reaction:


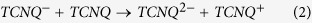


Existence of all five species, TCNQ, TCNQ^*^, TCNQ^−^, TCNQ^2−^, and TCNQ^+^ taking part in the above mechanism has been experimentally observed. Therefore, it is of high interest to explore this complex reaction scheme in a pure condensed phase containing only the strong quinone acceptors. This two-step process is triggered by a single-photon absorption with very low photon energy (2.6 eV).

In this work, we present the time-resolved spectroscopic study in TCNQ and F_4_TCNQ single crystals using femtosecond transient absorption (TA) and time-resolved fluorescence spectroscopy. Excited state dynamics reveals that after absorption of a single photon, dianions are formed in both crystals on an ultrashort timescale (<4 ps). We also demonstrated that the dianion formation is mediated by the radical anion precursor and described by a two-step model.

## Results

### Steady-state spectra and time-resolved fluorescence

**Figure 1:**
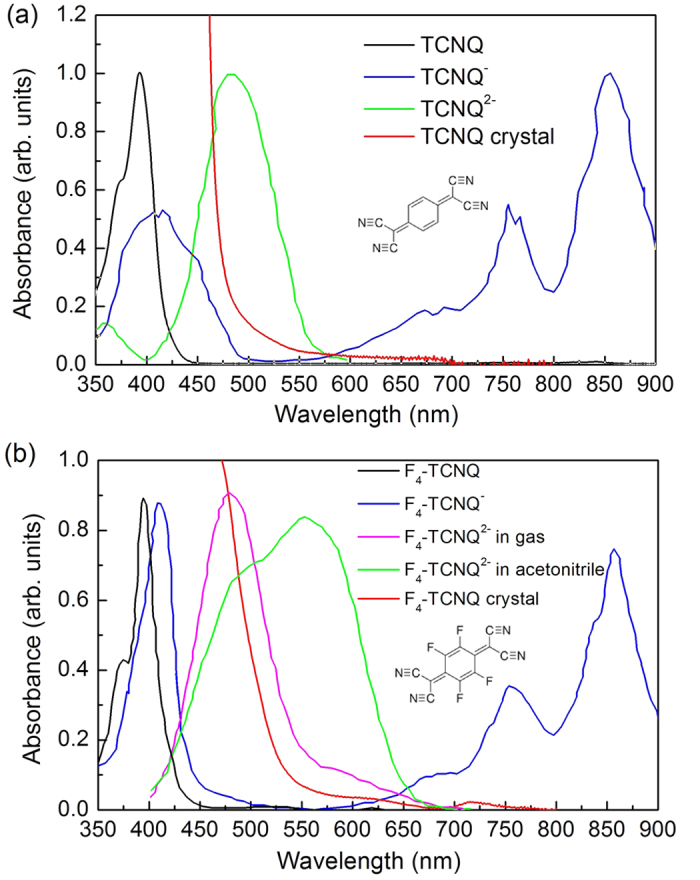
Steady-state absorption spectra of (**a**) TCNQ in acetonitrile, radical anion (TCNQ^•−^)^16^, dianion (TCNQ^2−^)^16^ and single crystal; (**b**) F_4_TCNQ in acetonitrile, radical anion (F_4_TCNQ^•−^)^17^, dianion (F_4_TCNQ^2−^) in acetonitrile^17^, dianion in gas^17^ and single crystal.

The steady-state absorption spectra of the neutral molecule, radical anion, dianion, and single crystals of TCNQ and F_4_TCNQ are shown in Fig. 1. Absorption maxima of neutral TCNQ and F_4_TCNQ in acetonitrile locate at 393 nm which correspond to transition S_0_ → S_1_. Due to the unpaired single electron in TCNQ and F_4_TCNQ anions, their electronic structure contains doublet states (labeled as D). F_4_TCNQ^•−^ and TCNQ^•−^ have similar absorption spectra: two main absorption bands, one at 410 nm corresponding to the D_0_ → D_2_ transition, and the other at 600–900 nm with two local maxima of 754 and 856 nm, due to the D_0_ → D_1_ transition^17^,^18^. In ref. 17, Panja *et al*. studied absorption and lifetime of the F_4_TCNQ^2−^ in the gas and solution. The major absorption band is located at 335 nm. Additional broad absorption in the 400–700 nm region is also observed both in the gas and solution. Both spectra can be described by the sum of two Gaussians (481 and 567 nm)^17^. However, in solution, the oscillator strength of 567 nm band is much stronger than that in the gas phase.

In our recent study^19^, we have characterized the spectroscopic properties of TCNQ in acetonitrile. The fluorescence recorded at room temperature is very weak (quantum yield *ϕ* < 10^−4^), with ultrafast decay time (800 fs). In the case of single crystals, both TCNQ and F_4_TCNQ crystals also do not show detectable steady-state fluorescence at room temperature. Their fluorescence lifetimes were measured by fluorescence upconversion technique (Fig. S2). There are two decay components in both crystals. In F_4_TCNQ, τ_1_ = 0.3–0.8 ps with amplitude about 70%, τ_2_ ranges from 4.5 to 12 ps with amplitude about 30% (Supplementary Table S1 and Fig. S2b). The ultrafast fluorescence indicates that there are efficient quenching channels as discussed below.

### Transient absorption spectra

**Figure 2:**
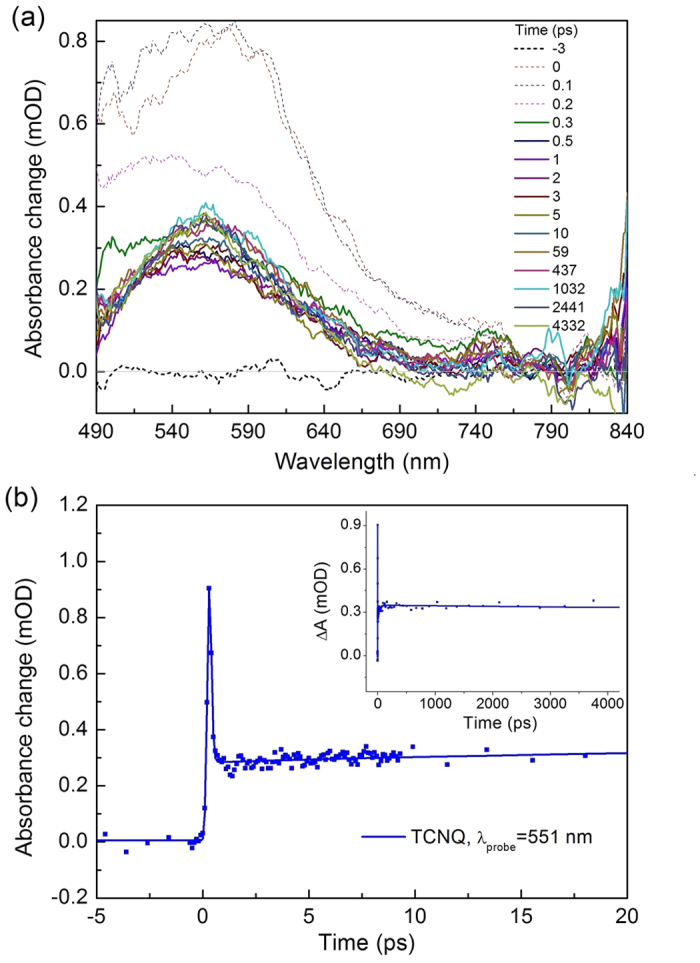
Transient absorption spectra (**a**) and kinetics (**b**) of TCNQ crystal, λ_*exc*_ = 475 nm.

**Figure 3:**
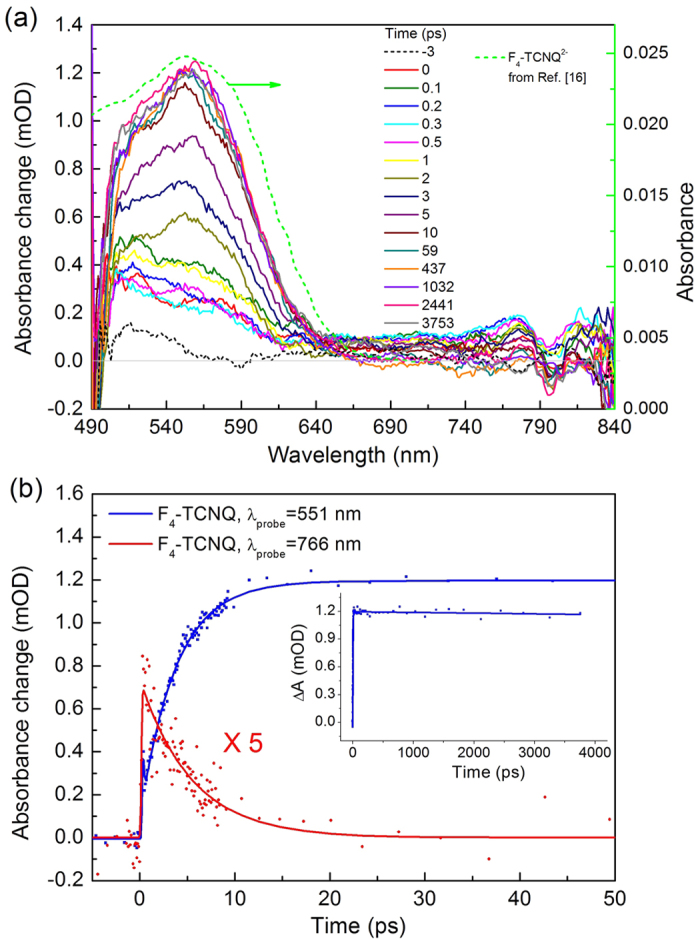
Transient absorption spectra (**a**) and kinetics at λ_*probe*_ = 551 nm (dianion) and 766 nm (radical anion) (**b**) in F_4_TCNQ crystal, λ_*exc*_ = 475 nm. The green dashed line in (**a**) corresponds to the absorption spectrum of F_4_TCNQ^2−^ in acetonitrile from ref. 17.

The transient absorption spectra of TCNQ and F_4_TCNQ crystals were measured under excitation at the absorption edge. The TA spectra and kinetics of TCNQ and F_4_TCNQ crystals under 475 nm excitation are presented in Figs 2 and 3, respectively. The main positive TA bands of both crystals are centered at 557 nm with very long lifetimes (no noticeable decay within 4 ns). In TCNQ crystal, this TA signal shows an instantanous rise, followed by an ultrafast decay with lifetime of 150 fs (Fig. 2b) which is comparable to the instrument response function (IRF) of the pump probe setup. This ultrafast process is due to nondegenerate two photon absorption (ND-TPA)^20^. ND-TPA is a process when one photon from the pump and one photon from probe beam are simultaneously absorbed by the sample, and thus the molecule appears in two-photon excited state. This process was observed only at λ_exc_ = 475 nm, i.e., in the absence of linear absorption. In the presence of strong one-photon absorption (excitation at 250 or 350 nm), this ultrafast process disappears (Supplementry Figs S5 and S6), i.e. no ND-TPA. In F_4_TCNQ crystal, the 557 nm TA band shows a slow rise (4.3 ps). Moreover, there is an additional weak TA band between 650 and 850 nm (Fig. 3a) which decays within 5 ps. This decay corresponds well with the rise of 557 nm TA band, indicating that one transient is transferred to the other. The origin of this transformation process is discussed below.

## Discussion

In F_4_TCNQ crystal, the peak position of the weak TA band between 650 and 850 nm (Fig. 3a) corresponds well with the absorption of radical anion F_4_TCNQ^•−^ (see Fig. 1b blue line). Therefore, we assign this band to the absorption of F_4_TCNQ^•−^. In Fig. 3a, for comparison, the absorption spectrum of F_4_TCNQ^2−^ in solution (Fig. 1b green line) is plotted together with our TA spectra. The location and shape of the 557 nm TA band are in excellent agreement with the absorption spectrum of F_4_TCNQ^2−^. In order to estimate the lifetime of this long-lived TA band, we also plotted the TA spectra at negative delay time (−3 ps), which can be considered as 1 ms time delay due to our 1 kHz laser repetition rate. We can see that the TA spectra at >550 nm show no visible decay within 4 ns but fully disappear within 1 ms, indicative of a lifetime being 4 ns << τ_1_ < 1 ms. However, in the probe range <550 nm, there is visible TA signal at negative delays. By a first order exponential fit, we can estimate this lifetime to be about 0.6 ms. This value also corresponds well with the reported F_4_TCNQ^2−^ lifetime of 0.7 ms (30%)^17^. The other two longer lifetimes of F_4_TCNQ^2−^ 15 ms (60%) and 1s (3%) reported in ref. 17 cannot be observed in our TA measurements due to the limitation of the 1 kHz laser. Therefore, we assign the TA band at 557 nm to the absorption of F_4_TCNQ^2−^.

As discussed above, the TA band between 650 and 850 nm (Fig. 3a) is due to the radical anion F_4_TCNQ^•−^, and 557 nm TA band is due to F_4_TCNQ^2−^. From the kinetics of both F_4_TCNQ^•−^and F_4_TCNQ^2−^ (Fig. 3b), we can see that the F_4_TCNQ^•−^ is generated instantaneously (<50 fs) and decays within 5 ps. Moreover, F_4_TCNQ^2−^ (557 nm transient) is formed slowly with a rise time of 4.3 ps; Therefore, we concluded that the radical anion F_4_TCNQ^•−^ is the precursor of F_4_TCNQ^2−^.

We also measured the TA spectra of F_4_TCNQ under excitation at 250 and 350 nm, shown in Figs S3 and S4 (Supplementry Information). Compared with the TA spectra under 475 nm excitation, they show the same 557 nm TA band but no visible F_4_TCNQ^•−^ absorption in 650–850 nm region. Moreover, in addition to the 4 ps rise time, an instantaneous rise component (<50 fs) appears in the TA kinetics. This additional ultrafast formation time under excitation at higher photon energies suggests that there is a competitive channel of dianion formation, i.e. direct two-electron transfer from the highly excited F_4_TCNQ to the unexcited neighboring molecules.

TCNQ can be considered as a analogue of F_4_-TCNQ, since their neutral and anion absorption spectra are very similar, which indicates the four fluoride substitudes do not remarkably distort the electronic structure. At time delays longer than 300 fs, the TA spectra of TCNQ crystal present a similar TA band at 557 nm, however, broader than that of F_4_TCNQ (Fig. 2a). This TA band is also long-lived without noticeable decay within 4 ns (Fig. 2b). Therefore, the 557 nm TA band in TCNQ crystal is also assigned to dianion (i.e., TCNQ^2−^). The TA kinetics of TCNQ^2−^ does not show any rise, indicative of an ultrafast dianion formation (<50 fs).

It is noteworthy that the anion absorption of TCNQ crystal is red shifted compared with TCNQ solution (Figs 1a and 2a) while the anion absorption from F_4_TCNQ crystal is similar to F_4_TCNQ solution (Figs 1b and 3a). We explain this due to the molecular packing difference in two crystals. In TCNQ crystal, adjacent TCNQ molecules pack in a cofacial manner (Fig. S1, Supplemental Material) which facilitates electron delocalization between neighbor TCNQs, resulting in a red shifted anion absorption. However, in F_4_TCNQ crystal, molecules pack in a fashion (Fig. S1, Supplemental Material) that the spatial overlap is poor between adjacent molecules. Therefore, the anion absorption in F_4_TCNQ crystal is less affected. A similar example can be found for NDI molecule which is also a well-known electron acceptor. In a cofacial stacking NDI dimer, the anion is more red shifted relative to the monomer anion^21^. However, in a triangular NDI prism where the cofacial overlap is much worse, the anion absorption spectrum is very similar to the NDI monomer solution^22^.

**Figure 4:**
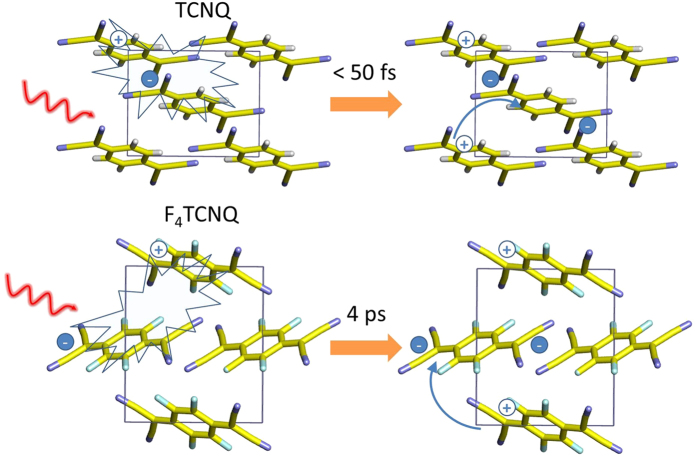
Two-step photogeneration of dianions in TCNQ and F_4_-TCNQ crystals.

The experimentally observed long absorption tails (Figs 2a and 3a) indicate the presence of a charge transfer transition in both TCNQ and F_4_TCNQ crystals, therefore, we explain the dianion formation mechanisms in TCNQ and F_4_TCNQ crystals with a two-step model: the initial excitation to a charge transfer excitation forms a cation and an anion, followed by a second step that is an electron transfer between the anion and an adjacent neutral molecule, forming a dianion. The dianion generation reaction scheme after band edge excitation of F_4_TCNQ is depicted in Fig. 4. Qualitativelly, the model and reactions for both TCNQ and F_4_TCNQ are similar. Within 50 fs after photoexcitation, charge transfer excitation results in a cation F_4_TCNQ^+^ and an anion F_4_TCNQ^•−^; then this F_4_TCNQ^•−^ within 4 ps adopts another electron from an adjacent neighbor F_4_TCNQ to form the double charged anion, i.e., dianion F_4_TCNQ^2−^. However, in TCNQ crystal, the second step is ultrafast, resulting in the absence of TCNQ anion intermediate in the TA spectra.

The molecular and electronic structures of TCNQ and F_4_TCNQ are very similar; the only apparent difference between TCNQ and F_4_TCNQ crystals is their crystalline structure: F_4_TCNQ crystal is orthorhombic while TCNQ crystal belongs to the monoclinic system, as shown in Supplementry Fig. S1. We can see that in TCNQ crystal, along the stacking direction, the neighbor molecules are well parallel to each other, i.e., they are in a stacking geometry with strong charge density overlap, resulting in a larger electronic coupling *H*_*el*_. However, in F_4_TCNQ, the molecular stacking is not as good as that of TCNQ, which will induce a poorer charge density overlap thus a smaller electronic coupling *H*_*el*_. Therefore, difference in rise times confirms that the dianion formation originates from the intermolecular interaction. It is in line with our conclusion that dianions are formed via electron transfer between neighboring molecules.

To sum up, in two strong organic electron acceptors TCNQ and F_4_TCNQ crystals, for the first time we observed a formation of single- and double-charged anions due to charge transfer between excited acceptor molecule and neutral acceptor molecules after absorption of one photon. In both crystals, after photon absorption, the anions are formed almost instantenously (within 50 fs). Next step includes formation of dianions by another electron uptake from the neighbor molecules. The dianion formation time is structure dependent: in the well packed monoclinic TCNQ crystal, TCNQ^2−^ forms within 50 fs. However, in the orthorhombic F_4_TCNQ crystal where the adjacent molecules are not well packed, F_4_TCNQ^2−^ forms much slower: within 4 ps. In the presence of different donor molecules, TCNQ and F_4_TCNQ may form charge transfer compounds with multiple stoichiometry. Such compounds have already been observed for perylene-TCNQ (1:1, 1:2, 1:3)^23^,^24^ but still not in perylene-F_4_TCNQ. Our work shows that in the absence of additional donor molecule, in the well packed strong acceptor systems photoexcitation leads to formation of doubly charged ions.

## Materials and Methods

### Materials

TCNQ and F_4_TCNQ commercial powders were purchased from Sigma-Aldrich. Their single crystals were grown by the physical vapor transport (PVT) technique^25^. The crystal structures of TCNQ and F_4_TCNQ were obtained using a Bruker SMART APEX II single crystal diffractometer (X-ray radiation, Mo Kα, λ  = 0.71073 Å). The fluorination makes F_4_TCNQ possessing a different crystalline structure from TCNQ: TCNQ crystal exhibits a monoclinic symmetry, and belongs to the C2/c space group with a = 8.865(2) Å, b = 6.883(1) Å, c = 16.387(1) Å, and 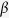 = 98.21(3)° 26. The F_4_TCNQ single crystal exhibits orthorhombic symmetry, and belongs to the Pbca space group with a = 9.300(8) Å, b = 8.19(1) Å, c = 14.628(6) Å.

### Absorption and fluorescence

Steady-state absorption spectra were obtained by a double beam UV-Vis spectrophotometer (Cary 100Bio, Varian). The fluorescence upconversion measurements were carried out by a system FOG 100 (CDP Systems). The second harmonic of a Titanium sapphire laser (Chameleon, Coherent Inc.) at 400 nm (100 fs, 80 MHz) was used as the excitation source. The fluorescence (430–650 nm) was collected by parabolic mirrors and focused onto a 0.5 mm BBO crystal (cut angle 38°, type I nonlinear interaction) together with the fundamental radiation (800 nm) to generate the sum-frequency radiation. The resulting radiation (280–359 nm) was detected by a photomultiplier based photon counting electronics after passing through a double monochromator (CDP2022D).

### Femtosecond laser spectroscopy

The transient absorption (TA) spectra were measured by the optical femtosecond pump probe technique. The output of titanium-sapphire (Legend Elite, Coherent) regenerative amplifier seeded by the oscillator (Micra, Coherent) was used as a pulse laser source. The output laser beam was with wavelength 800 nm, pulse width 65 fs, pulse repetition rate 1 kHz, and average power 3.5 W. This fundamental radiation was converted to 250–475 nm by use of optical parametric amplifier (Topas, Light Conversion); it was used as the pump beam. White light continuum generated in a sapphire plate was used as the probe beam^27^. The diameter of pump and probe beams are about 50 μm at focus point. The intensity ratio between pump and probe is about 20:1. The pump polarization is fixed at magic angle (54.7 degree) relative to the probe polarization (horizontal). The crystals were fixed at the edge of glass slides using double-sided adhesive with their long axes (b axes) parallel to the probe polarization (horizontal direction). The absorbance change 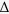
A is calculated by measuring the transmitted probe intensity in the presence (I_pump_on_) and in the absence (I_pump_off_) of the pump beam:


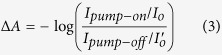


where I_0_ and I_0_′ are the respective reference signals. ΔA > 0 is indicative of excited state absorption (ESA), charge transfer states or formation of new species, and ΔA < 0 for ground state bleaching (GSB) or stimulated emission (SE). The spectral resolution is 1 nm, the signal resolution can reach 10^−4^ OD, i.e., 0.1 mOD. The time resolution is 50 fs after deconvolution with the instrument response function (IRF).

## Additional Information

**How to cite this article**: Ma, L. *et al*. Single photon triggered dianion formation in TCNQ and F_4_TCNQ crystals. *Sci. Rep*. **6**, 28510; doi: 10.1038/srep28510 (2016).

## Supplementary Material

Supplementary Information
