# Supplementary material for: Single photon triggered dianion formation in TCNQ and F4TCNQ crystals
Source: Sci Rep. 2016 Jun 27;6:28510. doi: 10.1038/srep28510 (PMC4921923; doi:10.1038/srep28510)
Supplement: Supplementary Information [file srep28510-s1.doc]

**Supplementary Information**

Single photon triggered dianion formation of TCNQ and F_4_TCNQ

Lin Ma,^1^ Peng Hu,^2^ Hui Jiang,^2^ Christian Kloc,^2^ Handong Sun,^1^ Cesare Soci,^1^ Alexander A. Voityuk,^3,4^ Maria E. Michel-Beyerle,^1^ and Gagik G. Gurzadyan^1,5*^

^1^ Division of Physics and Applied Physics, School of Physical and Mathematical Sciences, Nanyang Technological University, 637371, Singapore

*^2^ School of Materials Science and Engineering, Nanyang Technological University, 639798, Singapore*

*^3^ Instituciό Catalana de Recerca i Estudis Avançats (ICREA), Barcelona 08010, Spain*

*^4^ Institut de Química Computacional i Catàlisi (IQCC), Universitat de Girona, Campus de Montilivi 17071 Girona, Spain*

*^5^ Present address: Institute of Artificial Photosynthesis, State Key Laboratory of Fine Chemicals F-209, Dalian University of Technology, Dalian, 116024, China*

**Correspondence to gurzadyan@dlut.edu.cn or gurzadyan@ntu.edu.sg*

*
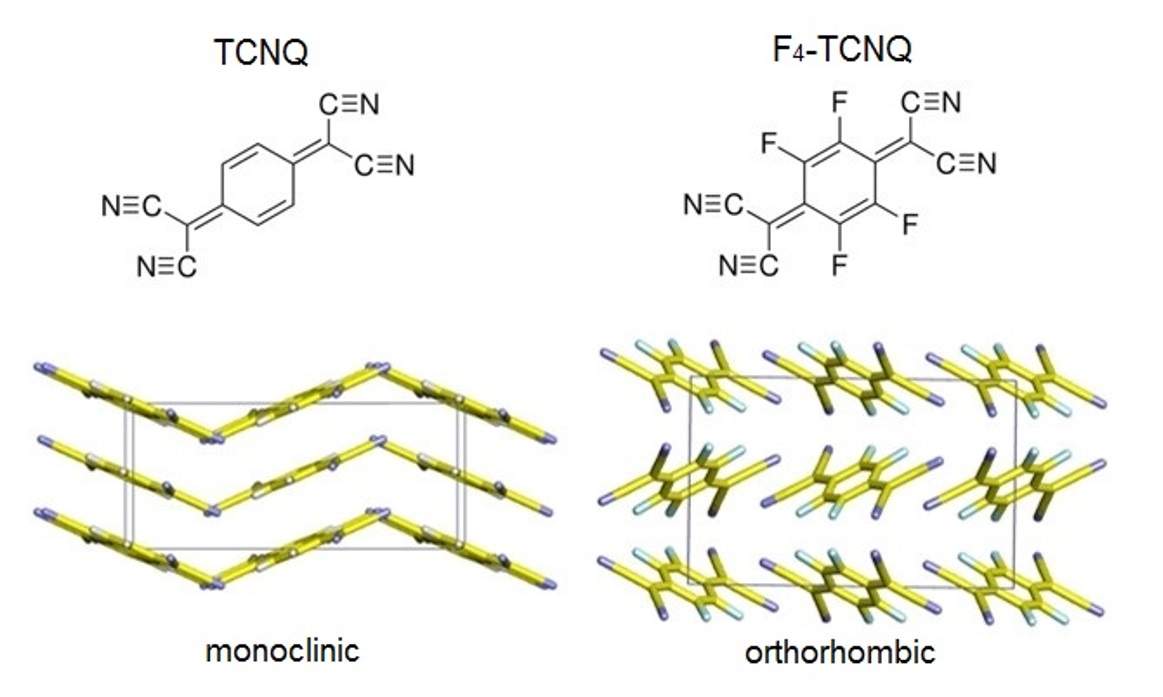
*

FIG S1. Molecular packing of TCNQ and F4-TCNQ.


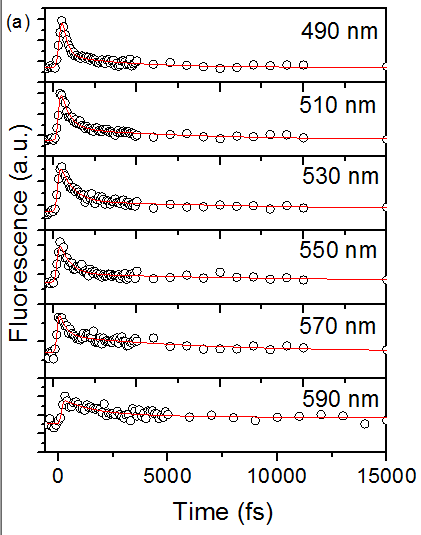

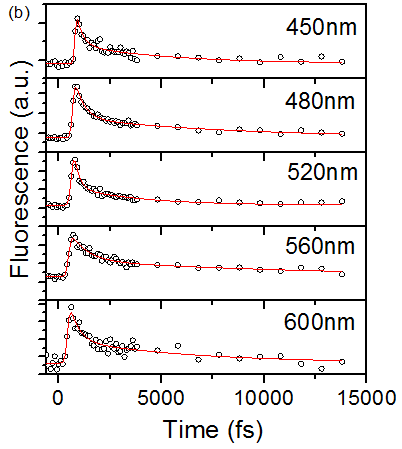


FIG S2. Fluorescence kinetics of TCNQ (a) and F_4_TCNQ (b) crystals, λ_exc_ = 400 nm.

TABLE S1. Fit results for the fluorescence kinetics of TCNQ and F_4_-TCNQ crystals.

| TCNQ | τ_1_, fs | A_1_ | τ_2_, fs | A_2_ | χ^2^ |
| --- | --- | --- | --- | --- | --- |
| 490 nm | 280 | 0.84 | 3800 | 0.26 | 0.973 |
| 510 nm | 520 | 0.80 | 7900 | 0.20 | 0.973 |
| 530 nm | 780 | 0.81 | 18000 | 0.19 | 0.959 |
| 550 nm | 630 | 0.82 | 17000 | 0.18 | 0.935 |
| 570 nm | 430 | 0.64 | 8900 | 0.36 | 0.882 |
| 590 nm | 1500 | 0.67 | 45000 | 0.33 | 0.822 |

| F_4_TCNQ | τ_1_, fs | A_1_ | τ_2_, fs | A_2_ | χ^2^ |
| --- | --- | --- | --- | --- | --- |
| 450 nm | 320 | 0.68 | 4500 | 0.32 | 0.930 |
| 480 nm | 450 | 0.70 | 7700 | 0.30 | 0.990 |
| 520 nm | 240 | 0.76 | 3200 | 0.24 | 0.975 |
| 560 nm | 830 | 0.65 | 12000 | 0.35 | 0.967 |
| 600 nm | 460 | 0.66 | 5900 | 0.34 | 0.895 |

*
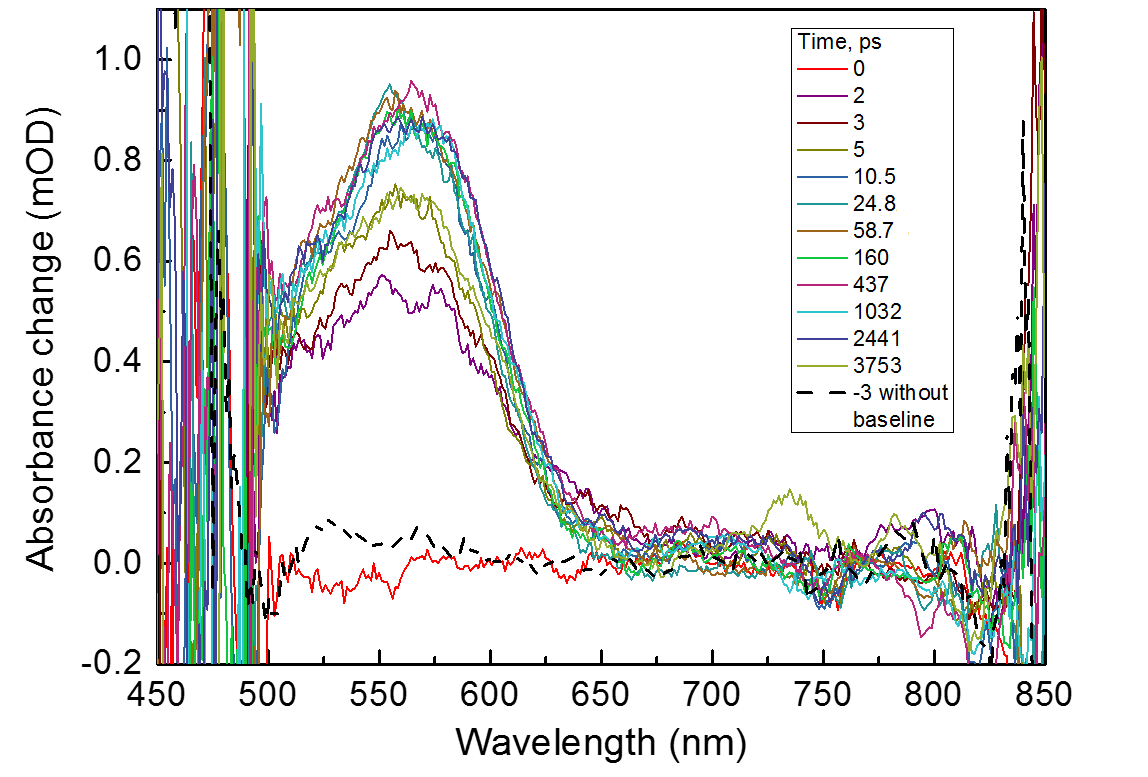
*

(b)

(a)


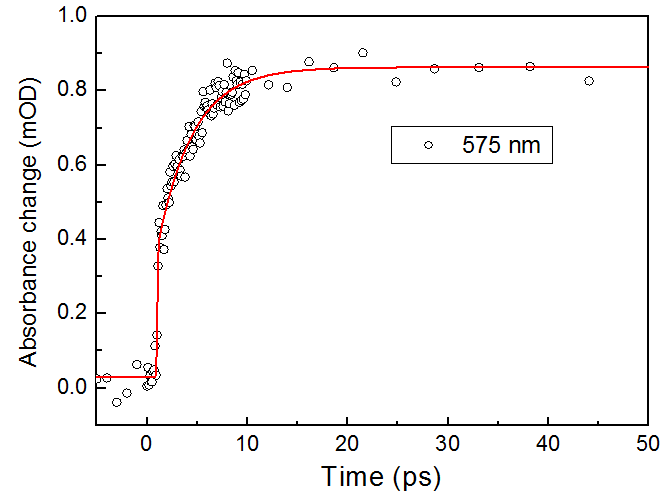


FIG S3. Transient absorption spectra (a) and kinetics (b) of F_4_-TCNQ crystal, λ_exc_ = 250 nm.

“-3 without baseline” indicates the trace is not subtracted by signal at negative delay time, while other traces are subtracted by the spectra at negative delay times to eliminate the influence from previous pump pulse.


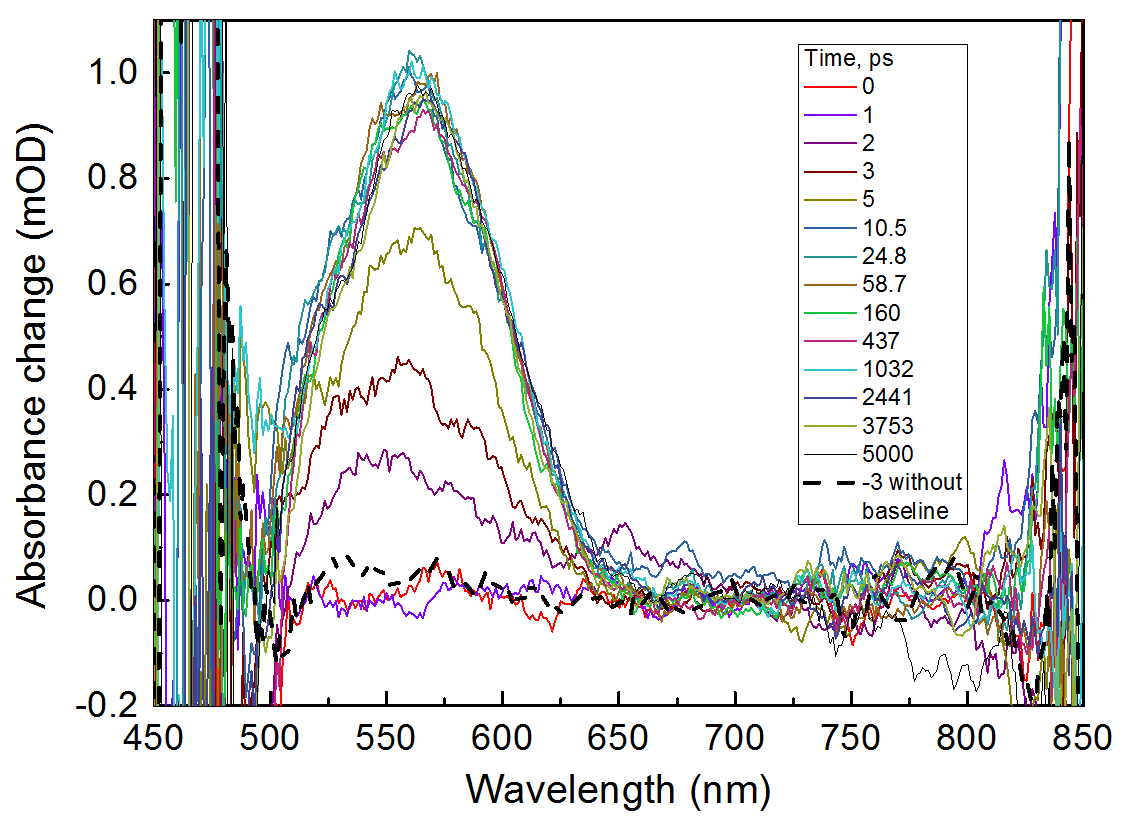


(a)

(b)


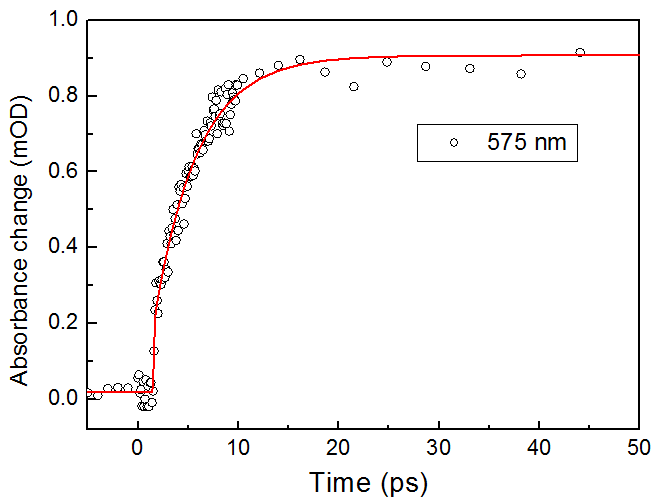


FIG S4. Transient absorption spectra (a) and kinetics (b) of F_4_-TCNQ crystal, λ_exc_ = 350 nm.


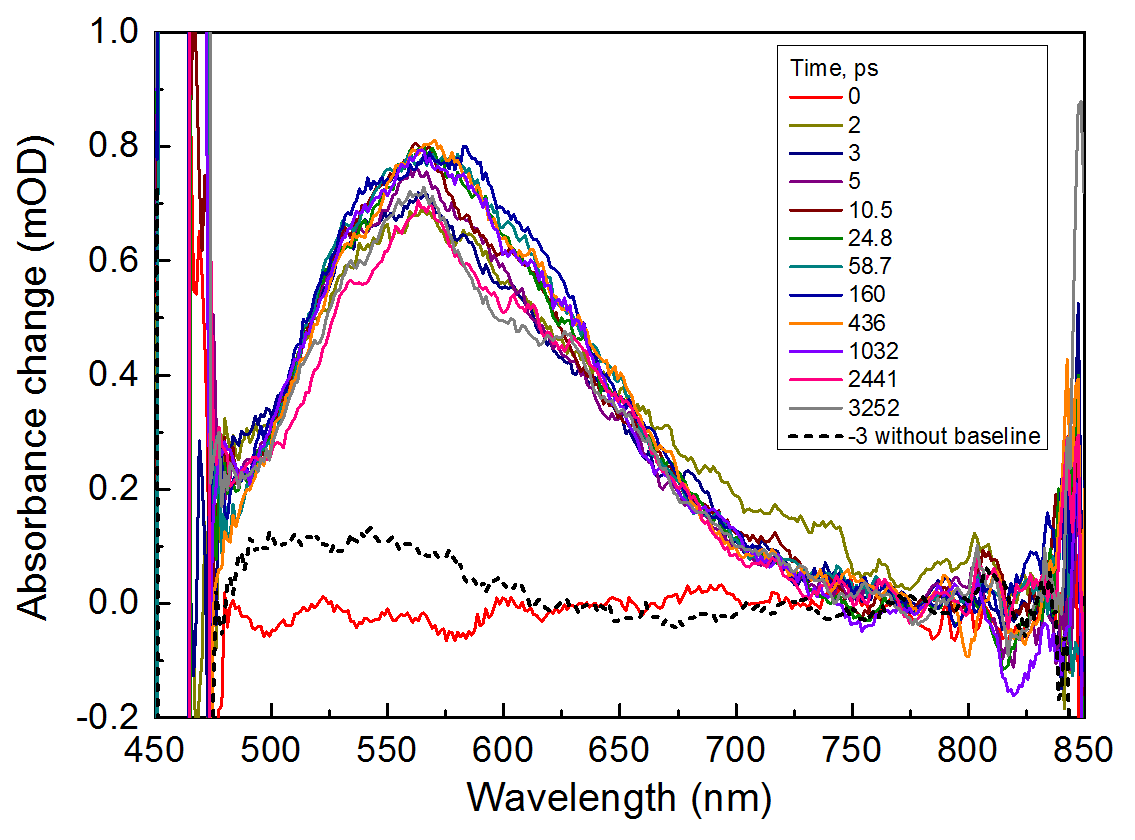


FIG S5. Transient absorption spectra of TCNQ crystal, λ_exc_ = 250 nm.


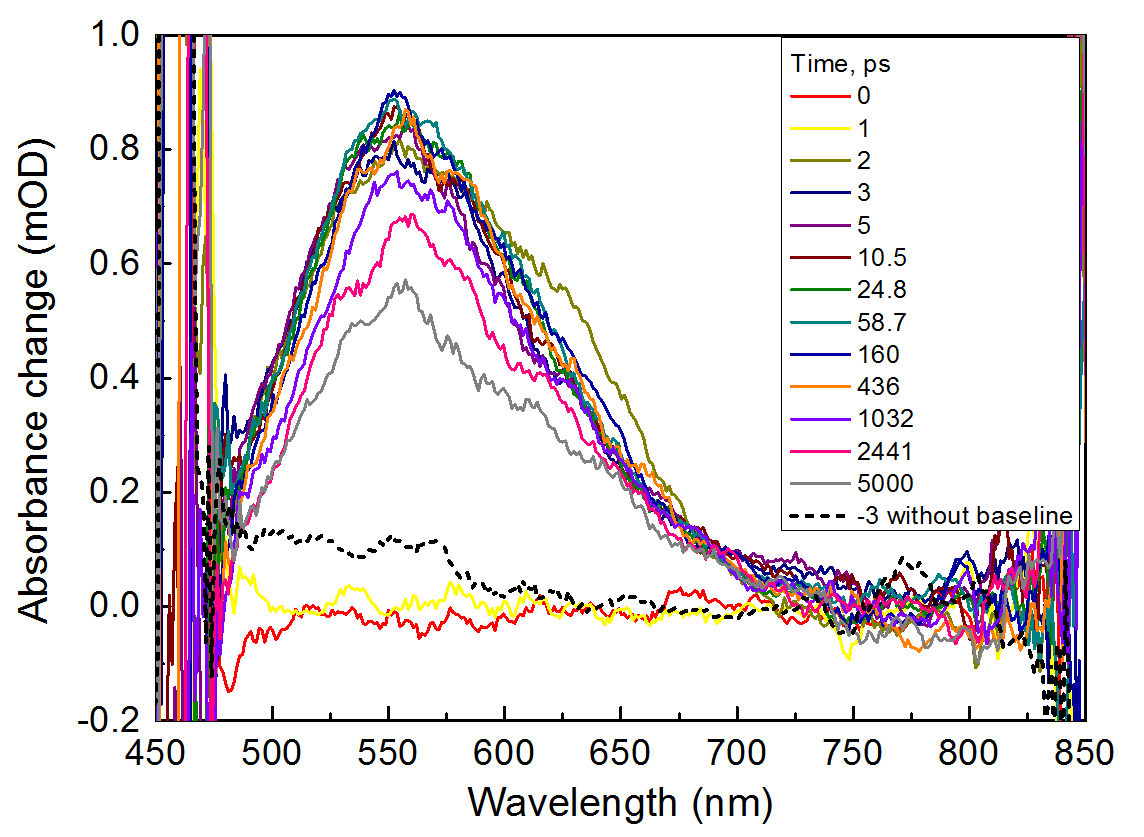


FIG S6. Transient absorption spectra of TCNQ crystal, λ_exc_ = 350 nm.
